# Development and radiographic evaluation of a normalised guide‐pin orientation abacus for anterior closing‐wedge tibial osteotomy: Comparison with fixed‐ratio and osteotomy‐depth methods

**DOI:** 10.1002/jeo2.70951

**Published:** 2026-10-09

**Authors:** Théo Plassard, Romir Patel, Christophe Jacquet, David Dejour, Antoine Piercecchi, Matthieu Ollivier

**Affiliations:** ^1^ Department of Orthopedic Surgery, APHM, CNRS, ISM, Institute of Movement Sciences, Sainte‐Marguerite Hospital Aix‐Marseille University Marseille France; ^2^ Department of Orthopaedic Surgery Massachusetts General Hospital Boston Massachusetts USA; ^3^ Lyon Ortho Clinic Clinique de la Sauvegarde Lyon France

**Keywords:** abacus, anterior closing‐wedge osteotomy, osteotomy planning, posterior tibial slope, slope‐reducing osteotomy, tibial osteotomy, trigonometric model

## Abstract

**Purpose:**

To develop a simplified mathematical model and abacus estimating anterior wedge resection height during slope‐reducing tibial osteotomy from patient‐specific guide‐pin length and normalised guide‐pin orientation, and to evaluate its accuracy under simulated radiographic conditions against fixed‐ratio and osteotomy‐depth methods. It was hypothesised that normalised guide‐pin orientation would provide wedge‐height estimates comparable to radiographic planning in both configurations.

**Methods:**

Two hundred fifty‐nine calibrated strict lateral knee radiographs from patients undergoing ACL reconstruction were retrospectively analysed in a single‐centre radiographic simulation study. Supratuberosity and infratuberosity slope‐reducing osteotomies were simulated with a standardised 10° correction, recording guide‐pin length, orientation and corresponding anterior wedge height. Three models were compared: a trigonometric model with normalised mean angles, a fixed‐ratio model and the osteotomy‐depth method. Accuracy (mean absolute error, 90th percentile error) was assessed in‐sample against radiographic reference measurements and confirmed by leave‐one‐out and five‐fold cross‐validation.

**Results:**

Mean guide‐pin orientation angles were 67.4° ± 6.0° (supratuberosity) and 110.6° ± 4.7° (infratuberosity). The normalised‐angle model produced mean absolute errors of 0.45 ± 0.41 mm (95% CI, 0.40–0.50) and 0.88 ± 0.79 mm (95% CI, 0.78–0.98), with 90th percentile errors of 0.91 and 1.77 mm and Bland–Altman bias of −0.17 and −0.06 mm, lower than both comparator models. Estimation error was not significantly influenced by sex or baseline posterior tibial slope, though the cohort's imbalanced sex distribution (194 men, 65 women) limited power to detect smaller effects in women.

**Conclusion:**

Within this model‐development cohort, a patient‐specific abacus based on guide‐pin length and normalised guide‐pin orientation produced low approximation errors under simulated radiographic conditions, without intraoperative radiographic calibration or complex calculations. These findings are exploratory and reflect in‐sample performance; independent and clinical validation, including prospective intraoperative testing, are required before the method can be considered accurate, reproducible or ready for intraoperative use.

**Level of Evidence:**

Level IV, retrospective radiographic simulation/model‐development study without clinical outcomes.

**Abbreviations**
A: angle between the supratuberosity guide pin and the anterior tibial cortex
ACL: anterior cruciate ligament
ANOVA: analysis of variance
ATT: anterior tibial tuberosity
B: angle between the infratuberosity guide pin and the anterior tibial cortex
Centricity™ Universal Viewer: digital radiographic viewing and measurement software
CI: confidence interval
cm: centimetre(s)
*d*
: Cohen's *d* effect size
et al.: and others
e.g.: for example
*F*
: *F*‐statistic
Fig.: figure
h: anterior wedge resection height for the supratuberosity osteotomy
h': anterior wedge resection height for the infratuberosity osteotomy
ICC: intraclass correlation coefficient
Inc.: incorporated
IQR: interquartile range
IRB: institutional review board
IT: infratuberosity or infratuberosity guide‐pin length
ITD: geometric label for the infratuberosity osteotomy triangle
JMP: JMP statistical software, version 20
Microsoft Excel: spreadsheet software
Min−Max: minimum–maximum
mm: millimetre(s)
M−W: men minus women
NC: North Carolina
*N*/*n*
: total sample size or subgroup number
*p*
: probability value
PCL: posterior cruciate ligament
Prob > *F*
: probability associated with an F‐statistic greater than the observed value
PTS: posterior tibial slope
*R* ^2^
: coefficient of determination
SAS: Statistical Analysis System/SAS Institute Inc.
SD: standard deviation
sin: sine function
ST: supratuberosity or supratuberosity guide‐pin length
STC: geometric label for the supratuberosity osteotomy triangle
TM/™: trademark
*t*‐test: Student's t‐test
USA: United States of America
vs.: versus
*x*
: multiplication operator in the displayed formula
<: less than
%: percentage
>: greater than
±: plus or minus
°: degree(s)
*α*
: alpha or statistical significance threshold
*β*
: regression coefficient
*θ*
: planned correction angle

## INTRODUCTION

Excessive posterior tibial slope (PTS) is a well‐established anatomical risk factor for anterior cruciate ligament (ACL) injury and graft failure [1, 9, 29], and slope‐reducing anterior closing‐wedge proximal tibial osteotomy has emerged as an effective adjunctive procedure for patients with excessive slope [2, 3, 15, 24, 27, 30]. The geometric relationship between osteotomy depth, wedge height and angular correction has been described using trigonometric models, first by Hernigou et al. [7, 8, 14], and osteotomy level and starting point significantly influence the required bone resection [12, 13].

Translating a planned angular correction into the corresponding anterior wedge resection intraoperatively remains challenging [25]. Simplified height‐per‐degree conversion ratios are commonly used despite reported variability across studies and configurations [3, 4, 21, 31], and osteotomy‐depth‐based methods that model the resection wedge as a symmetric (isosceles) triangle [8, 26] do not account for the orientation of the osteotomy plane relative to the anterior cortex, a factor that may be required to accurately model the asymmetric sagittal wedge [12, 28]. Fully individualised trigonometric calculation requires precise intraoperative measurement of guide‐pin orientation and osteotomy geometry, which may be difficult to perform reliably in routine surgical settings [10, 23, 25].

If guide‐pin orientation shows limited variability across patients, normalising this angle could allow a simplified, population‐derived model that retains trigonometric rigour while improving intraoperative applicability [11, 17]. The purpose of the present study was to develop a mathematical model and practical abacus based on normalised guide‐pin orientation for estimation of wedge height during anterior closing‐wedge tibial osteotomy, and to compare it with fixed‐ratio and osteotomy‐depth methods under simulated radiographic conditions. It was hypothesised that guide‐pin orientation follows reproducible geometric patterns amenable to normalisation, and that an abacus based on these normalised values would provide wedge‐height estimates comparable to radiographic planning within the model‐development cohort.

## MATERIALS AND METHODS

This was a retrospective, single‐centre, radiographic simulation study. Between January 2022 and December 2025, 634 lateral knee radiographs from patients undergoing primary ACL reconstruction at a single centre were assessed for eligibility (Figure 1).

**Figure 1:**
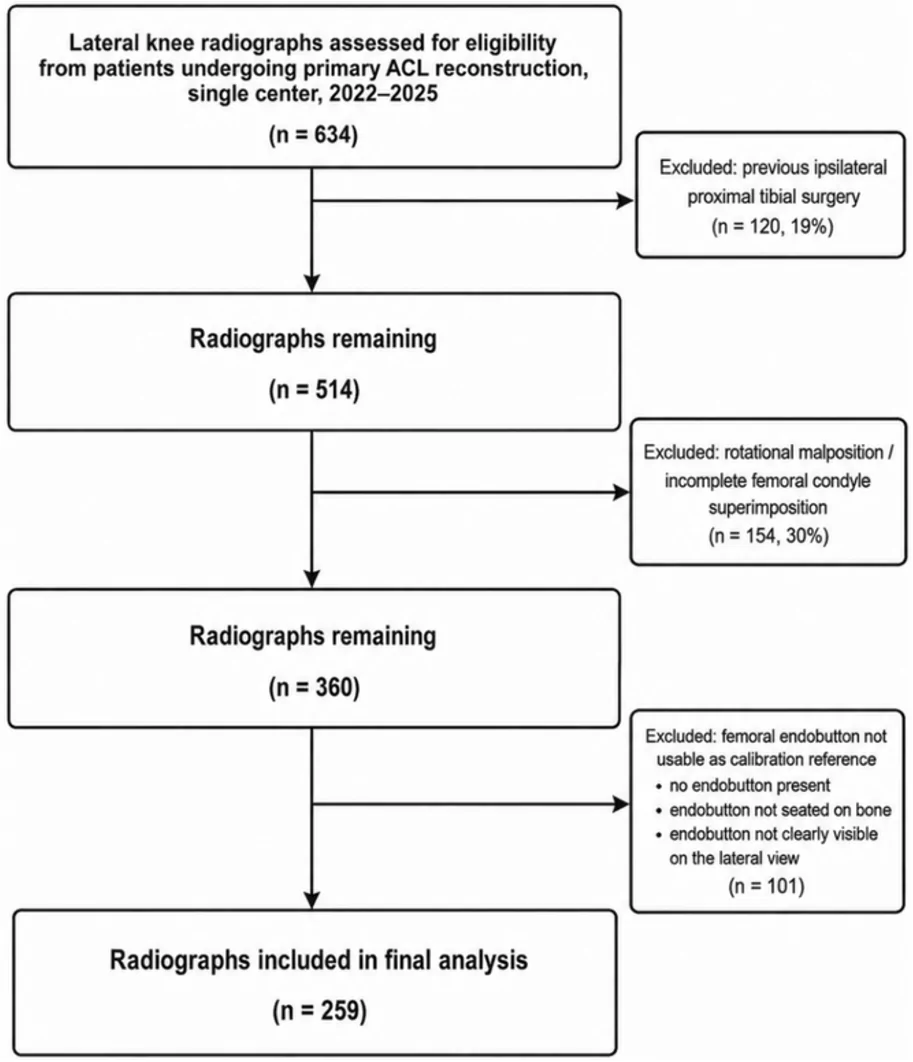
Radiograph selection flow diagram. Lateral knee radiographs from patients who underwent primary anterior cruciate ligament reconstruction between 2022 and 2025 were screened for eligibility. Radiographs were excluded for previous ipsilateral proximal tibial surgery, rotational malposition or incomplete superimposition of the femoral condyles or absence of a clearly visible and appropriately seated femoral endobutton suitable for calibration. A total of 259 radiographs were included in the final analysis. ACL, anterior cruciate ligament.

This study received Institutional Review Board approval (IRB #Cse_pads25_043_dgr).

Radiographs were excluded for previous ipsilateral proximal tibial surgery, rotational malposition or incomplete femoral condyle superimposition, or an unusable femoral endobutton, which served as the calibration reference for digital measurement (absent, not seated on bone or not clearly visible on the lateral view). After exclusions, 259 radiographs met all quality criteria and were included in the final analysis.

Radiographs were selected based on strict quality criteria, including true lateral projection with satisfactory superimposition of the femoral condyles and adequate visualisation of the proximal tibial metaphysis, as recommended in previous morphometric studies [5, 12, 19]. A radiograph was considered a true lateral (‘strict’) projection when the posterior offset between the medial and lateral femoral condyles did not exceed 5 mm and any apparent discrepancy in tibial slope attributable to rotational malpositioning did not exceed 5 mm, consistent with previously validated criteria; [5, 18, 19] radiographs failing these criteria were excluded, as detailed in the selection flow diagram (Figure 1) [18, 19]. Each patient contributed a single lateral radiograph (the operative knee) to the analysis; the dataset contained no duplicate or bilateral entries per patient.

All radiographs were digitally calibrated using standardised methods to ensure accurate measurements, consistent with previously described protocols [12, 21]. Calibration used the known length of the femoral cortical (interference) endobutton (14 mm) as an internal scale reference, with digital correction for radiographic magnification; an endobutton was accepted as a calibration reference only when fully seated on bone and unambiguously visualised in profile on the lateral view (Figure 1). The potential influence of endobutton position relative to the image receptor and tibia on scaling accuracy was not separately quantified and is addressed in the limitations.

### Radiographic planning and measurements

Slope‐reducing anterior closing‐wedge osteotomies were simulated on each radiograph according to established geometric principles [19], based on the trigonometric relationships described by Hernigou et al. linking wedge height, osteotomy length and hinge position [7, 8]. Two configurations were analysed: supratuberosity and infratuberosity osteotomies [20], reflecting common clinical practice [3, 16, 17, 27]. A standardised correction of 10° was applied in all cases to allow consistent comparisons [12, 21]. A single 10° correction angle was selected because it approximates a commonly targeted clinical correction for slope‐reducing osteotomy and permitted an unconfounded, controlled comparison of the three predictive models against a common angular target. Because the trigonometric relationship underlying the normalised‐angle model is a continuous function of the correction angle (Appendix S1), wedge‐height values for angles other than 10° in the accompanying abacus (Appendices 3–4, which span 3°–14°) are mathematically derived from this relationship but were not independently simulated or empirically evaluated; the same applies to guide‐pin lengths outside the observed range of this cohort (41.9–79.9 mm supratuberosity; 46.7–87.7 mm infratuberosity), even though the tabulated abacus range is 30–110 mm. These extrapolated regions—which include the extreme correction and length values most likely to carry the greatest clinical consequence if inaccurate—should be used with caution pending targeted validation (see Limitations).

Osteotomy geometry was defined using simulated guide pins. For supratuberosity osteotomy, the first guide pin was the inferior one, positioned from the summit of the anterior tibial tuberosity (ATT) to the posterior hinge at 1 cm from the tibial insertion of the posterior cruciate ligament, while the second pin was proximal and converged toward the hinge, forming a 10° angle with the first pin. For infratuberosity osteotomy, the first guide pin was the superior one, positioned 2 cm below the summit of the ATT, and the second guide pin was distal and also converged to the hinge point with a 10° angle [7, 8, 16, 27]. This configuration generated a triangular wedge allowing direct measurement of the required anterior resection height (Figure 2).

**Figure 2:**
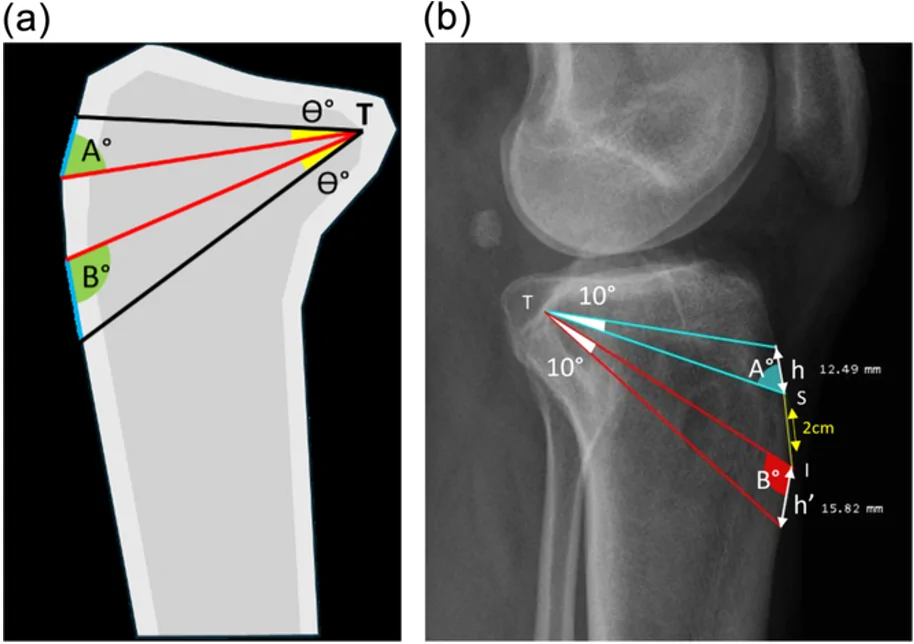
Geometric construction (a) and radiographic simulation (b) of supratuberosity and infratuberosity anterior closing‐wedge osteotomies. (a) Schematic geometric construction on a sagittal projection of the proximal tibia. The supratuberosity osteotomy wedge is defined by triangle STC, where ST represents the guide‐pin length, A represents the angle between the guide pin and the anterior tibial cortex, *h* represents the anterior wedge resection height, and *θ* represents the planned correction angle. The infratuberosity osteotomy wedge is defined by triangle ITD, with corresponding parameters IT, B, h′, and θ. (b) Corresponding radiographic simulation. (Blue) Supratuberosity osteotomy simulation. (Red) Infratuberosity osteotomy simulation. *h*, height of the resection wedge for a 10° supratuberosity correction; h′, height of the resection wedge for a 10° infratuberosity correction; A°, angle between the supratuberosity guide pin (ST) and the anterior cortex; B°, angle between the infratuberosity guide pin (IT) and the anterior cortex. ITD, geometric label for the infratuberosity osteotomy triangle; ST, supratuberosity for supratuberosity/infratuberosity osteotomy; STC, geometric label for the supratuberosity osteotomy triangle.

Measurements were performed on calibrated radiographs using digital software (Centricity™ Universal Viewer). Guide‐pin length and orientation relative to the anterior cortex were recorded, along with the corresponding anterior wedge height. The radiographic landmarks and measurements used for simulation are illustrated in Figure 2.

All measurements were performed by a single primary observer. Intraobserver reliability was assessed by having the primary observer repeat all measurements in a randomly selected subset of 50 radiographs during a second reading session performed 2 weeks after the first, to minimise recall bias. Interobserver reliability was assessed by having a second, independent observer, blinded to the primary observer's values, measure the same 50‐radiograph subset once. Both the primary and secondary observers were senior orthopaedic surgeons with more than 2 years of clinical experience following residency. Intraclass correlation coefficients (ICC) with 95% confidence intervals were calculated for guide‐pin length, guide‐pin angle and wedge height (Table 1).

**Table 1:** Intra and interobserver reliability (intraclass correlation coefficients).

Measure	Intraobserver ICC [95% CI]	Interobserver ICC [95% CI]
Height h (or h′)	0.82 [0.77–0.86]	0.74 [0.68–0.79]
Guide‐pin length (IT/ST)	0.79 [0.74–0.84]	0.71 [0.65–0.77]
Angle (ST/IT)	0.85 [0.81–0.89]	0.78 [0.73–0.82]

*Note*: ICC, intraclass correlation coefficient with 95% confidence interval. Intraobserver reliability was assessed by repeat measurements from the same observer; interobserver reliability was assessed across independent observers.

Abbreviations: IT, infratuberosity; h/h′, anterior wedge resection height; ST, supratuberosity for supratuberosity/infratuberosity osteotomy.

Three predictive models were evaluated. The first was a trigonometric model using patient‐specific guide‐pin length combined with normalised mean guide‐pin angles derived from the study population, based on the assumption of reproducible geometric patterns [8, 12, 21] (Appendix S1). The second was a fixed‐ratio model using a constant wedge height per degree of correction, based on the measured height of the study population (1° = 1 mm for supratuberosity and 1° = 1.4 mm for infratuberosity osteotomies), as previously proposed [3, 4, 21]. The third model was added for comparison with the recently published osteotomy‐depth method described by Vieider et al. [26], and wedge height was calculated using their proposed formula of 2 × sin(0.5 × 10°) × osteotomy depth, with the guide‐pin length corresponding to the osteotomy depth.

Based on the best‐performing simplified model, an abacus—a tabular reference chart allowing the required wedge height to be read directly from two measured inputs (guide‐pin length and target correction angle) without calculation—was constructed to allow intraoperative estimation of wedge height using guide‐pin length and correction angle [8, 12, 21].

Prediction accuracy was assessed by comparing model‐derived wedge heights with reference values measured on radiographic simulation. Absolute error was calculated as the absolute difference between predicted and reference values [12, 21]. Mean absolute error, standard deviation and percentile distributions were reported. For each tested model, the predicted wedge height was converted into an angular‐equivalent correction using the inverse of the trigonometric formula derived from the patient‐specific radiographic geometry (Appendix S2). Absolute difference from the planned 10° correction was reported as angular error, and the proportion of simulations remaining within ±1° of the planned correction was also calculated.

Explanatory variables included guide‐pin length and orientation (ST and A for supratuberosity, IT and B for infratuberosity), measured wedge heights (h and h′) and PTS (Figure 2). Associations were analysed using univariate linear regression, reported as *β* coefficients, *R* ^2^ and *p*‐values.

Statistical analysis was performed using JMP (version 20; SAS Institute Inc.). Data were prepared using Microsoft Excel. Results are presented as mean absolute error, standard deviation, median and interquartile range, with the 90th percentile used to assess clinical accuracy. Model‐predicted wedge heights were compared with radiographic reference measurements using two‐tailed paired Student's *t*‐tests. The influence of sex was analysed using one‐way ANOVA, with 95% confidence intervals and Cohen's *d* reported. Associations between estimation error and morphometric variables were analysed using univariate linear regression and reported as *β* coefficients, *R* ^2^ and *p*‐values. Statistical significance was set at *p* < 0.05. For measures reported as mean ± SD, 95% confidence intervals were additionally derived as mean ± 1.96 × (SD/√N); 95% confidence intervals for proportions were derived using the Wilson score method. Confidence intervals for the 90th‐percentile error and root‐mean‐square error (RMSE) were derived by case‐level bootstrap resampling (5000 resamples with replacement) of the original dataset. Proportional bias in the Bland–Altman analyses was formally tested by linear regression of the difference (predicted—measured) on the mean of predicted and measured values. To assess whether the reported in‐sample errors were optimistic, the normalised‐angle model was additionally evaluated by leave‐one‐out and five‐fold cross‐validation: for each held‐out patient (or fold), the normalised mean guide‐pin angle was recomputed using only the remaining patients and applied to predict the held‐out patient's wedge height.

## RESULTS

A total of 259 calibrated strict lateral radiographs were included in the analysis. Both supratuberosity and infratuberosity osteotomy configurations were simulated on each radiograph. The mean osteotomy length measured along the guide pin was 60.1 ± 5.8 mm for supratuberosity osteotomies and 69.0 ± 6.1 mm for infratuberosity osteotomies. The corresponding mean anterior wedge heights required to achieve a standardised 10° slope correction were 10.9 ± 1.2 mm and 13.9 ± 1.6 mm, respectively (Table 2).

**Table 2:** Demographic characteristics and morphometric measurements.

Parameters	Mean ± SD (*n* = 259, men = 194, women = 65)	Min–Max
Age (years)	28.6 ± 9.2	15.0–61.0
Tibial slope (°)	9.1 ± 3.3	0.0–17.4
ST distance (mm)	60.1 ± 5.8	41.9–79.9
Angle A (ST) (°)	67.4 ± 6.0	46.9–80.1
Height h (ST) (mm)	10.9 ± 1.2	7.5–14.9
IT distance (mm)	69.0 ± 6.1	46.7–87.7
Angle B (IT) (°)	110.6 ± 4.7	94.3–121.8
Height h′ (IT) (mm)	13.9 ± 1.6	9.2–19.6

*Note*: Values are presented as mean ± standard deviation with range (minimum–maximum).

Abbreviations: IT, infratuberosity guide‐pin length; Angle A, angle between the supratuberosity guide pin and the anterior tibial cortex; Angle B, angle between the infratuberosity guide pin and the anterior tibial cortex; h, anterior wedge resection height for supratuberosity osteotomy; h′, anterior wedge resection height for infratuberosity osteotomy; ST, supratuberosity guide‐pin length.

Guide‐pin orientation angles demonstrated comparatively narrow dispersion across patients. The mean angle relative to the anterior tibial cortex was 67.4° ± 6.0° (range, 46.9°–80.1°) for supratuberosity osteotomies and 110.6° ± 4.7° (range, 94.3°–121.8°) for infratuberosity osteotomies (Table 2).

The simplified trigonometric model using normalised mean guide‐pin angles (67° for supratuberosity; 110° for infratuberosity; Appendix S1) was evaluated against radiographic reference measurements within the same cohort from which the normalised angles were derived (in‐sample evaluation).

For supratuberosity osteotomies, the mean absolute error was 0.45 ± 0.41 mm (95% CI, 0.40–0.50), with a median of 0.34 mm [IQR, 0.15–0.67] and a 90th percentile of 0.91 mm (bootstrap 95% CI, 0.86–1.08; RMSE 0.60 mm, 95% CI, 0.54–0.67). For infratuberosity osteotomies, the mean absolute error was 0.88 ± 0.79 mm (95% CI, 0.78–0.98), with a median of 0.63 mm [IQR, 0.37–1.22] and a 90th percentile of 1.77 mm (bootstrap 95% CI, 1.59–2.07; RMSE 1.18 mm, 95% CI, 1.02–1.34).

To assess whether these in‐sample errors were optimistic, the normalised‐angle model was re‐evaluated by leave‐one‐out cross‐validation, recomputing the normalised mean angle from the remaining 258 patients for each held‐out case. Cross‐validated performance was essentially unchanged from the in‐sample estimate: mean absolute error 0.45 ± 0.41 mm (95% CI, 0.40–0.50; median 0.34 mm, 90th percentile 0.92 mm) for supratuberosity and 0.88 ± 0.78 mm (95% CI, 0.79–0.98; median 0.68 mm, 90th percentile 1.73 mm) for infratuberosity. A five‐fold cross‐validation gave concordant results (fold‐level mean absolute errors ranging from 0.38 to 0.50 mm for supratuberosity and from 0.78 to 1.06 mm for infratuberosity). Because a population mean estimated from 258 to 259 patients changes minimally when one patient is withheld, this indicates that the reported errors are not an artifact of in‐sample evaluation.

Bland–Altman analysis showed a mean signed bias of −0.17 mm (95% CI, −0.24 to −0.10) with 95% limits of agreement of −1.31 to 0.97 mm (95% CI of limits, −1.43 to −1.19 and 0.85 to 1.09) for supratuberosity, and a bias of −0.06 mm (95% CI, −0.20 to 0.08) with limits of agreement of −2.37 to 2.25 mm (95% CI of limits, −2.62 to −2.12 and 2.00 to 2.50) for infratuberosity (Figure 3). A formal test for proportional bias (linear regression of the difference on the mean of predicted and measured values) was statistically significant for both configurations (supratuberosity: slope −0.14 mm per mm, *p* < 0.0001; infratuberosity: slope −0.31 mm per mm, *p* < 0.0001), indicating that prediction error became more negative (greater overestimation by the model) as true wedge height increased; this proportional bias was not visually obvious on the Bland–Altman plots and should be taken into account when interpreting model performance across the range of wedge heights, particularly for larger infratuberosity resections. Individual observations fell outside the 95% limits of agreement in 15/259 (5.8%) supratuberosity and 13/259 (5.0%) infratuberosity simulations, close to the ~5% statistically expected outside 95% limits of agreement; the largest deviations reached −2.12 to 2.11 mm (supratuberosity) and −5.47 to 2.39 mm (infratuberosity). The clinical implications of these outliers for an individual osteotomy are addressed in the Discussion.

**Figure 3:**
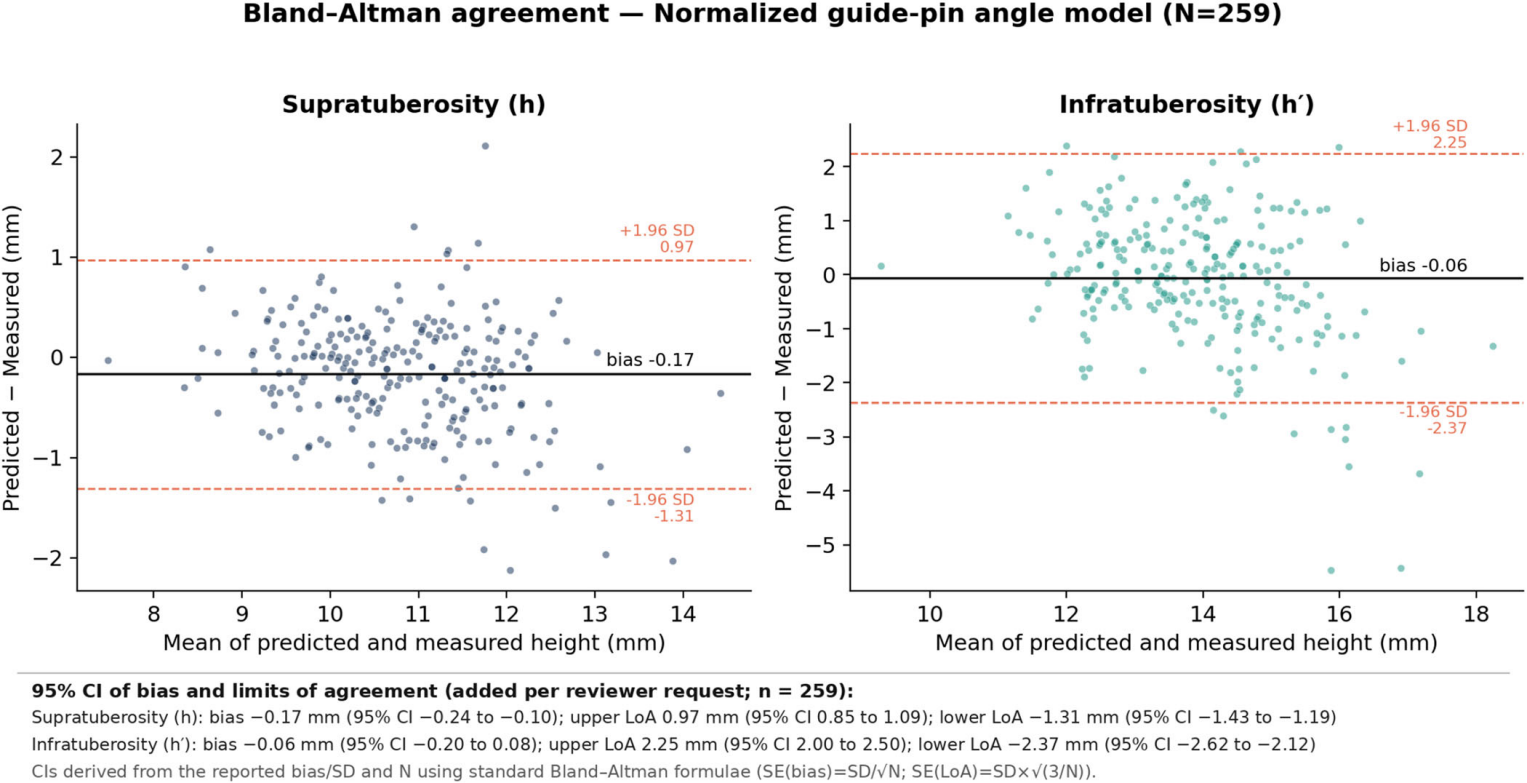
Bland–Altman agreement plots for the normalised guide‐pin angle model, supratuberosity (left) and infratuberosity (right) configurations (*N* = 259). Bias and 95% limits of agreement are shown with their own 95% confidence intervals (shaded bands), derived from the reported bias, SD, and *N* using standard Bland–Altman formulae. CI, confidence interval; SD, standard deviation.

The difference between this model and measured height reached statistical significance only for the supratuberosity configuration (*p* < 0.0001), whereas no significant difference was observed for infratuberosity osteotomies (*p* = 0.4087).

In contrast, empirical fixed‐ratio models showed greater prediction errors. The 1° = 1 mm model (supratuberosity) demonstrated a mean absolute error of 1.17 ± 0.90 mm (95% CI, 1.06–1.28), with a median of 0.90 mm [IQR, 0.40–1.75] and a 90th percentile of 2.30 mm, significantly different from reference values (*p* < 0.0001). The 1° = 1.4 mm model (infratuberosity) demonstrated a mean absolute error of 1.26 ± 0.99 mm (95% CI, 1.14–1.38), with a median of 1.10 mm [IQR, 0.50–1.70] and a 90th percentile of 2.40 mm, without reaching statistical significance (*p* = 0.29). Fixed‐ratio models demonstrated 2.6‐fold (supratuberosity) and 1.4‐fold (infratuberosity) greater mean absolute errors than the normalised‐angle model (Table 3).

**Table 3:** Model performance.

Config.	Model	MAE ± SD (mm)	95% CI of MAE (mm)	Median [IQR] (mm)	90th pct (mm) [95% CI]	RMSE (mm) [95% CI]	Angular error ± SD (°) [95% CI]	Within ± 1° (%) [95% CI]
ST	Normalised‐angle (*A* = 67°)	0.45 ± 0.41	0.40–0.50	0.34 [0.15–0.67]	0.91 [0.86–1.08]	0.60 [0.54–0.67]	0.20 ± 0.21 [0.17–0.23]	98.1 [95.6–99.2]
	1°/1 mm (fixed‐ratio)	1.17 ± 0.90	1.06–1.28	0.90 [0.40–1.75]	2.30 [2.10–2.42]	1.48 [1.34–1.61]	1.00 ± 0.71 [0.91–1.09]	54.4 [48.4–60.4]
	Osteotomy‐depth	0.54 ± 0.46	0.48–0.60	0.42 [0.19–0.76]	1.10 [1.06–1.26]	0.71 [0.64–0.78]	0.28 ± 0.28 [0.25–0.31]	96.9 [94.0–98.4]
IT	Normalised‐angle (*B* = 110°)	0.88 ± 0.79	0.78–0.98	0.63 [0.37–1.22]	1.77 [1.59–2.07]	1.18 [1.02–1.34]	0.35 ± 0.28 [0.32–0.38]	97.3 [94.5–98.7]
	1°/1.4 mm (fixed‐ratio)	1.26 ± 0.99	1.14–1.38	1.10 [0.50–1.70]	2.40 [2.20–2.84]	1.60 [1.44–1.76]	0.64 ± 0.46 [0.58–0.70]	82.2 [77.1–86.4]
	Osteotomy‐depth	1.88 ± 1.15	1.74–2.02	1.80 [1.09–2.42]	3.23 [2.97–3.55]	2.20 [2.03–2.38]	1.29 ± 0.37 [1.24–1.34]	21.2 [16.7–26.6]

*Note*: Values are presented as mean absolute error ± standard deviation, 95% confidence interval, median with interquartile range, 90th percentile, RMSE, angular error and proportion of simulations within ±1° of the planned correction, each with 95% CI (mean‐based CI for continuous measures; Wilson score CI for proportions; case‐level bootstrap resampling, 5000 resamples, for the 90th percentile and RMSE), *N* = 259. P‐values for paired comparisons with radiographically measured reference values are reported in the main text; all reached *p* < 0.0001 except the infratuberosity normalised‐angle (*p* = 0.4087) and 1°/1.4 mm models (*p* = 0.29).

Abbreviations: IT, infratuberosity; MAE, mean absolute error; RMSE, root‐mean‐square error; ST, supratuberosity.

The osteotomy‐depth method showed a mean absolute error of 0.54 ± 0.46 mm (95% CI, 0.48–0.60) for supratuberosity osteotomies, with a median error of 0.42 mm [IQR, 0.19–0.76], a 90th percentile error of 1.10 mm and a statistically significant difference compared with radiographic reference measurements (*p* < 0.0001). For infratuberosity, the mean absolute error was 1.88 ± 1.15 mm (95% CI, 1.74–2.02), with a median error of 1.8 mm [IQR, 1.09–2.42], a 90th percentile error of 3.23 mm and a significant difference (*p* < 0.0001). Relative to the normalised‐angle model, the osteotomy‐depth method showed 1.2‐fold (supratuberosity) and 2.1‐fold (infratuberosity) greater mean absolute errors.

For the angular‐equivalent errors, the normalised guide‐pin orientation model showed lower errors in both supratuberosity and infratuberosity configurations, with mean errors of 0.20° ± 0.21° (95% CI, 0.17°–0.23°) and 0.35° ± 0.28° (95% CI, 0.32°–0.38°), respectively, compared with 1.00° ± 0.71° (95% CI, 0.91°–1.09°) and 0.64° ± 0.46° (95% CI, 0.58°–0.70°) for the fixed‐ratio models, and 0.28° ± 0.28° (95% CI, 0.25°–0.31°) and 1.29° ± 0.37° (95% CI, 1.24°–1.34°) for the osteotomy‐depth method. The normalised guide‐pin orientation model remained within ±1° of the planned correction in 98.1% (95% CI, 95.6%–99.2%) supratuberosity simulations and 97.3% (95% CI, 94.5%–98.7%) infratuberosity simulations. The corresponding proportions were 54.4% (95% CI, 48.4%–60.4%) and 82.2% (95% CI, 77.1%–86.4%) for the fixed‐ratio models, and 96.9% (95% CI, 94.0%–98.4%) and 21.2% (95% CI, 16.7%–26.6%) for the osteotomy‐depth method.

The normalised‐angle trigonometric model showed lower absolute errors than both fixed‐ratio models and the osteotomy‐depth method, particularly in the infratuberosity configuration.

Direct pairwise comparisons confirmed this ranking statistically (Table 4). For supratuberosity osteotomies, the normalised‐angle model produced significantly lower error than the fixed‐ratio model (mean difference −0.72 mm, 95% CI, −0.82 to −0.62, *p* < 0.0001) and than the osteotomy‐depth method (−0.09 mm, 95% CI, −0.11 to −0.06, *p* < 0.0001), while the osteotomy‐depth method was itself significantly more accurate than the fixed‐ratio model (+0.63 mm, *p* < 0.0001). For infratuberosity osteotomies, the normalised‐angle model was significantly more accurate than both the fixed‐ratio model (−0.38 mm, 95% CI, −0.49 to −0.27, *p* < 0.0001) and the osteotomy‐depth method (−1.01 mm, 95% CI, −1.14 to −0.87, *p* < 0.0001); the osteotomy‐depth method performed worse than the fixed‐ratio model in this configuration (difference −0.63 mm, *p* < 0.0001). All six pairwise comparisons remained significant after Bonferroni correction (adjusted *α* = 0.0083) and were corroborated by two‐tailed Wilcoxon signed‐rank tests. This ranking was consistent with the mean absolute error comparison (Figure 4).

**Table 4:** Head‐to‐head comparison of predictive models—paired analysis of absolute error (*N* = 259).

Comparison	Mean diff (mm)	95% CI (mm)	Paired *t*‐test *p*	More accurate model
Normalised‐angle versus Fixed‐ratio (ST)	−0.72	−0.82 to −0.62	<0.0001*	Normalised‐angle
Normalised‐angle versus Osteotomy‐depth (ST)	−0.09	−0.11 to −0.06	<0.0001*	Normalised‐angle
Fixed‐ratio versus Osteotomy‐depth (ST)	+0.63	0.54 to 0.73	<0.0001*	Osteotomy‐depth
Normalised‐angle versus Fixed‐ratio (IT)	−0.38	−0.49 to −0.27	<0.0001*	Normalised‐angle
Normalised‐angle versus Osteotomy‐depth (IT)	−1.01	−1.14 to −0.87	<0.0001*	Normalised‐angle
Fixed‐ratio versus Osteotomy‐depth (IT)	−0.63	−0.78 to −0.47	<0.0001*	Fixed‐ratio

*Note*: Mean difference = mean (error of first‐named model)—mean (error of second‐named model); negative values indicate the first‐named model is more accurate. Paired two‐tailed Student's *t*‐tests were performed on paired absolute errors. All six comparisons remained significant after Bonferroni correction (adjusted *α* = 0.0083) and were corroborated by two‐tailed Wilcoxon signed‐rank tests (all *p* < 0.0001). Asterisk denotes statistical significance (*p* < 0.05).

Abbreviations: IT, infratuberosity; ST, supratuberosity.

**Figure 4:**
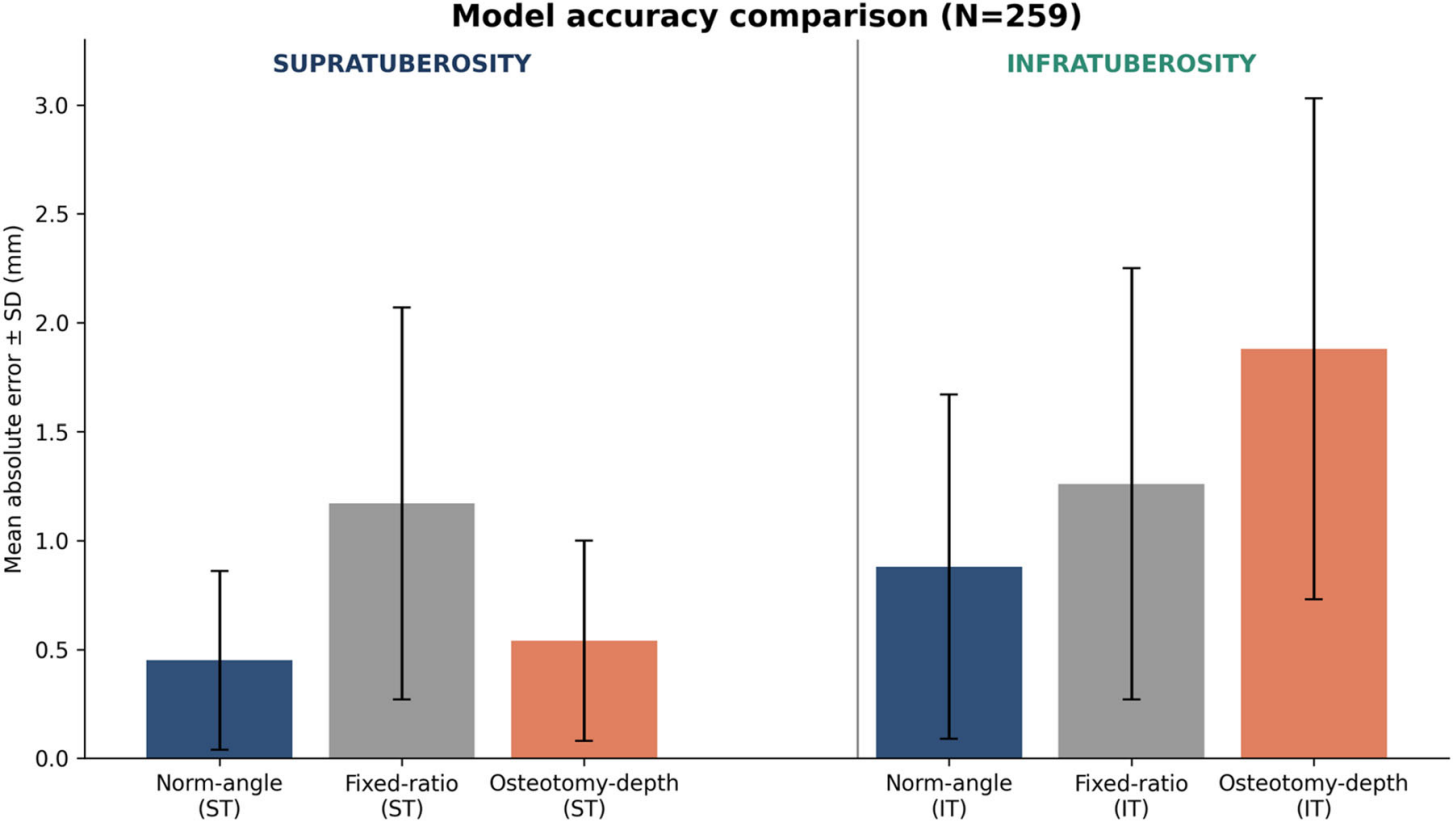
Mean absolute error (± SD) comparison across all three predictive models, supratuberosity and infratuberosity configurations (*N* = 259). IT, infratuberosity or infratuberosity guide‐pin length; SD, standard deviation.

Based on the simplified normalised‐angle model, correction tables (abacus) were constructed for both supratuberosity (Appendix S3) and infratuberosity (Appendix S4) osteotomies, providing the required wedge resection height according to guide‐pin length and target correction angle.

No significant association was observed between estimation error using the normalised‐angle and osteotomy‐depth models and patient sex for either the supratuberosity or infratuberosity configurations (Table S1, Supplementary Material). In contrast, fixed‐ratio models showed significant sex‐related differences in estimation error (*p* < 0.0001 for the 1° = 1 mm model; *p* = 0.03 for the 1° = 1.4 mm model). Given the cohort's sex distribution (194 men, 65 women), statistical power to detect a smaller effect in women was limited; this nonsignificant result does not exclude a clinically relevant sex‐related effect (see Limitations). Bivariate regression analysis (Table S2, Supplementary Material) showed that estimation error using normalised angles was not significantly influenced by PTS for either osteotomy configuration.

## DISCUSSION

The most important finding of the present study is that guide‐pin orientation during anterior closing‐wedge proximal tibial osteotomy demonstrates limited interindividual variability and follows reproducible geometric patterns. Based on this observation, normalisation of guide‐pin orientation allows accurate prediction of wedge height using a simplified trigonometric model for both supratuberosity and infratuberosity slope‐reducing osteotomy. Compared with fixed‐ratio models and with the recently described osteotomy‐depth method of Vieider et al., the proposed model showed lower absolute error, particularly in the infratuberosity configuration. These findings suggest that osteotomy depth alone may not be sufficient to estimate the geometry of the anterior resection wedge, which is a nonisosceles triangle, without considering the orientation of the osteotomy plane relative to the anterior tibial cortex, as suggested in a previous study [12]. These findings enabled the development of a practical abacus that allows estimation of wedge height based solely on guide‐pin length and planned angular correction, pending clinical validation.

A central finding of this study is the consistency of guide‐pin orientation across patients, with mean values of 67° for supratuberosity and 110° for infratuberosity osteotomies. These values remained stable regardless of patient sex or PTS, suggesting that osteotomy geometry is constrained by reproducible anatomical and technical factors rather than individual morphological variation. This observation is clinically important because it provides the foundation for normalisation: if guide‐pin angles were highly variable, a population‐derived mean would introduce unacceptable prediction error. The narrow distributions observed in the present study support that normalisation is feasible within this cohort; whether it remains reliable in other populations, surgeons, institutions and radiographic techniques has not been tested (see Limitations).

The geometric relationship between osteotomy length, wedge height and correction angle has been well described in classical mathematical models. Hernigou et al. [7, 8] established the theoretical foundation for osteotomy planning, whereas Quinn et al. [21] proposed empirical ratios to simplify the conversion of angular correction into wedge height. However, such fixed‐ratio approaches do not account for individual osteotomy geometry and, as confirmed by the present results, demonstrate substantially greater prediction errors than trigonometric models. Kayaalp et al. [12] further demonstrated that osteotomy level and orientation significantly influence both wedge geometry and cortical mismatch, reinforcing the need for individualised geometric considerations.

Vieider et al. recently proposed a simplified osteotomy‐depth method for infratuberosity osteotomy, which represents an important step away from fixed height‐per‐degree ratio models [26]. However, this approach approximates the resection wedge as two congruent right‐angled triangles forming an isosceles triangle and does not incorporate the orientation of the osteotomy plane. In the present cohort, this method performed reasonably in the supratuberosity configuration but underestimated wedge height for the infratuberosity configuration, supporting the relevance of including guide‐pin orientation in the predictive model.

The present study extends these observations by showing that the geometric variability requiring individualisation lies primarily in guide‐pin length rather than guide‐pin angle, and that the angle component can be normalised with limited loss of accuracy compared with the other models studied [12].

The simplified trigonometric model demonstrated mean absolute errors below 1 mm for both osteotomy configurations, with 90th percentile errors of 0.91 and 1.77 mm for supratuberosity and infratuberosity osteotomies, respectively. The clinical significance of errors of this magnitude has not been independently established and should be interpreted cautiously [12, 21]. Although the difference between the trigonometric model and radiographic reference reached statistical significance for supratuberosity osteotomies, the absolute magnitude of this difference—a bias of −0.17 mm (95% CI, −0.24 to −0.10)—is distinct from, and substantially smaller than, the mean absolute error of 0.45 mm, which reflects the average magnitude of error irrespective of direction; this bias is unlikely to carry clinical significance and likely falls within the precision limits of intraoperative measurement. For the infratuberosity configuration, the paired difference was not significant (bias −0.06 mm, 95% CI, −0.20 to 0.08); here, the wider 95% limits of agreement (−2.37 to 2.25 mm) are more informative than this null result about the range of error an individual patient might experience. In contrast, the fixed‐ratio models not only showed larger mean errors but also demonstrated significant sex‐related variability in prediction accuracy, a limitation not observed with the trigonometric approach. These findings underscore the importance of maintaining a geometric framework while simplifying model inputs.

From a practical perspective, the normalisation approach translates complex trigonometric relationships into a simple intraoperative tool. By combining guide‐pin length—which the surgeon can measure directly during surgery—with a population‐derived normalised guide‐pin angle, wedge height can be estimated without the need for intraoperative radiographic calibration, angular measurement of the guide pin or complex calculations. This concept led to the development of an abacus (Appendices 3 and 4) that provides direct correspondence between guide‐pin length, correction angle and required wedge resection height.

The proposed intraoperative workflow assumes that guide‐pin length can be measured directly from the anterior cortex to the intended osteotomy hinge—for example, using a graduated instrument or the guide‐pin itself—and that hinge position can be reliably identified and maintained, typically under fluoroscopic guidance. Saw blade thickness, the precise reference points used to define resection height, and rounding or interpolation between tabulated abacus values may each introduce additional error not captured in this radiographic simulation. These practical sources of variability should be characterised in a prospective clinical study before the method is described as accurate or reproducible for intraoperative use.

Accurate wedge height determination is critical because both undercorrection and overcorrection carry clinical consequences. Persistent excessive slope may result in continued graft overload and recurrent instability, while overcorrection may lead to altered joint kinematics, increased posterior tibial translation, or genu recurvatum [3, 24, 27]. The abacus proposed in this study addresses this challenge by providing a reference that preserves the mathematical rigour of trigonometric planning while remaining accessible in routine surgical settings, pending the clinical validation discussed above.

It is worth noting that the abacus is complementary to, rather than a replacement for, emerging technologies such as three‐dimensional patient‐specific instrumentation or robotic‐assisted osteotomy systems [10], which offer additional precision but require specific infrastructure and resources that may not be universally available. Its clinical role relative to patient‐specific digital preoperative planning, which is already used at many centres and allows individualised consideration of osteotomy level, hinge position and tibial morphology, also remains to be defined; the present study did not test whether the abacus adds accuracy beyond digital planning. It is proposed as a low‐cost, equipment‐independent intraoperative cross‐check or as an alternative where digital planning is unavailable, rather than as a replacement for individualised digital planning—particularly for patients whose anatomy deviates from the population mean, for whom patient‐specific planning may be preferable given the outlier magnitudes noted above. The abacus provides an accessible alternative that can be used in any operating room without specialised equipment, particularly in complex corrections like biplanar osteotomy where small technical deviations can affect the final correction [6].

The choice of ±1° as the threshold for acceptable angular accuracy was adopted as a pragmatic benchmark consistent with the precision typically reported for freehand and image‐based osteotomy execution [22, 31], rather than a threshold with an established causal link to clinical outcome. The clinical consequence of angular errors of this magnitude on postoperative slope, ACL graft strain or reoperation has not been established in this study and should be examined prospectively. Similarly, statistically significant differences between models—particularly the submillimetric differences reported here—are unlikely to exceed the precision of intraoperative measurement and surgical execution and should not be over‐interpreted as clinically distinguishing.

The present findings are consistent with and extend the geometric principles described by Hernigou et al. [7, 8], by demonstrating that guide‐pin orientation can be normalised across patients, thereby bridging the gap between theoretical planning and practical surgical execution.

Several limitations should be acknowledged. First, the study is based on radiographic simulations rather than intraoperative measurements. Although such simulations are commonly used in osteotomy research and allow precise and reproducible geometric analysis [12, 21], intraoperative variability in guide‐pin placement, soft tissue constraints and saw blade thickness may introduce additional error not captured in this model. Clinical validation with intraoperative measurements and postoperative slope assessment is warranted. Second, a standardised correction of 10° was applied to all simulations and was not independently simulated at other angles; although the trigonometric relationships identified in this study are mathematically applicable across different correction angles, the prediction accuracy at other corrections—including the extreme values covered by the abacus (3°–14°)—was not empirically validated and may differ, particularly since extreme corrections are potentially associated with the greatest clinical consequence of error. Third, guide‐pin positioning was simulated according to standard surgical technique rather than measured intraoperatively, which may not capture the full range of variability encountered in clinical practice. Fourth, the normalised angle values (67° and 110°) were derived from a single‐centre population. Their performance was primarily evaluated in the same cohort used to derive them (in‐sample/apparent performance); leave‐one‐out and 5‐fold cross‐validation, in which the normalised angle was recomputed from the remaining patients for each held‐out case, produced essentially unchanged error estimates, indicating that the in‐sample result is not an artifact of evaluating the model on its own derivation cohort. This cross‐validation nonetheless remains internal to a single‐centre population; external validation in different populations, surgeons, institutions and radiographic techniques is still required before generalisability beyond this cohort can be assumed. Fifth, the analysis was performed on two‐dimensional lateral radiographs, which do not account for the three‐dimensional geometry of the proximal tibia, including rotational or oblique osteotomy planes.

Sixth, calibration relied on the femoral cortical button as an internal scale reference; the position of this implant relative to the image receptor and the tibia may influence scaling accuracy, and this source of error was not separately quantified. Seventh, the sex distribution of the cohort was imbalanced (194 men, 65 women); the absence of a statistically significant sex effect on estimation error does not exclude a smaller, clinically relevant effect in the underrepresented female subgroup, for which the study was comparatively underpowered. Eighth, radiographs were obtained from patients undergoing primary ACL reconstruction, whereas anterior closing‐wedge slope‐reducing osteotomy is more commonly considered in the revision ACL setting; the cohort's mean PTS (9.1°, range 0°–17.4°) may therefore not fully represent the population in whom this osteotomy is typically performed, and the normalised angles should be interpreted with this selection bias in mind. Finally, claims of reproducibility in this manuscript refer to measurement reliability (Table 1) and to the internal mathematical consistency of the geometric model, not to prospectively demonstrated intraoperative accuracy, which has not yet been tested.

Despite these limitations, the large sample size allowed robust evaluation of guide‐pin orientation variability, cross‐validation supported the stability of the normalised‐angle estimates within this cohort, and the consistency observed across demographic subgroups supports the use of normalised geometric parameters within this population. Future studies should validate the abacus in a clinical setting by comparing predicted wedge heights with intraoperative measurements and postoperative slope correction, and assess whether the normalised angle values are applicable across different surgical centres and patient populations.

## CONCLUSION

Within this model‐development cohort, a patient‐specific abacus based on guide‐pin length and normalised guide‐pin orientation produced low approximation errors under simulated radiographic conditions, without intraoperative radiographic calibration or complex calculations. These findings are exploratory and reflect in‐sample performance; independent and clinical validation, including prospective intraoperative testing, are required before the method can be considered accurate, reproducible or ready for intraoperative use.

## AUTHOR CONTRIBUTIONS

All authors contributed to the study conception (Matthieu Ollivier, David Dejour) or design (Théo Plassard, Christophe Jacquet, Antoine Piercecchi, Romir Patel). Material preparation, data collection (Théo Plassard) and analysis (Matthieu Ollivier and Romir Patel) were performed at APHM. The first draft of the manuscript was written by Théo Plassard, and all authors commented on previous versions of the manuscript. All authors read and approved the final manuscript.

## CONFLICT OF INTEREST STATEMENT

David Dejour has received royalties from Arthrex, Science & BioMaterials (SBM), and Corin; and consulting fees from Smith & Nephew. Matthieu Ollivier receives consulting fees and royalties from Stryker Corporation and Newclip Technologies. All other authors have no disclosures.

## ETHICS STATEMENT

Institutional approval was obtained before study initiation. IRB #Cse_pads25_043_dgr.

## Supporting information

Appendices 1–4 (mathematical derivations and the full sagittal correction abaci for supratuberosity and infratuberosity osteotomy) and Supplementary Tables S1–S2 (sex‐stratified and bivariate regression analyses) are provided in the accompanying Supplementary File.

## Data Availability

All data used in this study are available from the corresponding author upon reasonable request.
