# Supplementary material for: Development and radiographic evaluation of a normalised guide‐pin orientation abacus for anterior closing‐wedge tibial osteotomy: Comparison with fixed‐ratio and osteotomy‐depth methods
Source: J Exp Orthop. 2026 Oct 9;13(4):e70951. doi: 10.1002/jeo2.70951 (PMC13652586; doi:10.1002/jeo2.70951)
Supplement: Supplementary file 1 [file JEO2-13-e70951-s001.docx]

**Supplementary File**

*Development and Radiographic Evaluation of a Normalized Guide-Pin Orientation Abacus for Anterior Closing-Wedge Tibial Osteotomy: Comparison with Fixed-Ratio and Osteotomy-Depth Methods*

This file contains Appendices 1–4 and Supplementary Tables S1–S2 referenced in the main manuscript.

**APPENDICES**

**Appendix 1. Mathematical derivation of the equations governing the trigonometric models**

In a two-dimensional sagittal projection of the proximal tibia, the supratuberosity osteotomy wedge is represented by triangle STC.
ST: length of the guide pin. SC = h: resection height measured on the anterior cortex. A: angle between segment ST and the tangent to the anterior cortex. θ: planned sagittal correction angle, opposite side SC.
The sum of the angles in a triangle is equal to 180°. In triangle STC, angle C is therefore: C = 180 − (A + θ). According to the law of sines: h / sin(θ) = ST / sin(C). Thus: h = ST × sin(θ) / sin(C) = ST × sin(θ) / sin(180 − (A + θ)). Since sin(180 − x) = sin(x), it follows that h = ST × sin(θ) / sin(A + θ). The same derivation applies to triangle ITD (infratuberosity osteotomy wedge): h′ = IT × sin(θ) / sin(B + θ).

**Appendix 2. Mathematical derivation of the inverse equation for angular-equivalent correction**

Solving the relationship h = ST × sin(θ) / sin(A + θ) for θ: h sin(A + θ) = ST sin(θ); since sin(A + θ) = sin(A)cos(θ) + cos(A)sin(θ): h sin(A) = [ST − h cos(A)] tan(θ); θ = arctan[h sin(A) / (ST − h cos(A))]. The same derivation applies to triangle ITD: θ = arctan[h′ sin(B) / (IT − h′ cos(B))].

Appendix 3 and Appendix 4. Sagittal correction abaci for supratuberosity and infratuberosity slope-reducing osteotomy (unchanged; full tables retained from the original manuscript file). Rows/columns corresponding to guide-pin lengths outside the observed range of this cohort (< 41.9 mm or > 79.9 mm for supratuberosity; < 46.7 mm or > 87.7 mm for infratuberosity) and to correction angles other than 10° represent mathematical extrapolation and were not independently validated;

**SUPPLEMENTARY TABLES**

**Supplementary Table S1. Influence of sex on resection height estimation error across the different models**

| **Configuration / Model** | **Women (n=65) MAE±SD** | **Men (n=194) MAE±SD** | **Diff (M−W), mm** | **95% CI (mm)** | **p / Cohen's d** |
| --- | --- | --- | --- | --- | --- |
| ST — Normalized-angle (A=67°) | 0.42 ± 0.33 | 0.46 ± 0.43 | 0.03 | −0.07 to 0.13 | 0.5523 / 0.085 |
| ST — 1°/1 mm model | 0.72 ± 0.57 | 1.32 ± 0.94 | 0.60 | 0.41 to 0.80 | <0.0001* / 0.699 |
| ST — Osteotomy-depth | 0.49 ± 0.36 | 0.55 ± 0.49 | 0.06 | −0.05 to 0.17 | 0.386 / 0.124 |
| IT — Normalized-angle (B=110°) | 0.74 ± 0.63 | 0.92 ± 0.85 | 0.18 | −0.02 to 0.37 | 0.1205 / 0.223 |
| IT — 1°/1.4 mm model | 1.49 ± 0.88 | 1.18 ± 1.02 | −0.32 | −0.57 to −0.06 | 0.0257* / −0.32 |
| IT — Osteotomy-depth | 1.83 ± 0.95 | 1.90 ± 1.21 | 0.07 | −0.22 to 0.35 | 0.6811 / 0.06 |

Values are mean absolute difference ± SD for women (n=65) and men (n=194), with mean difference (men minus women), 95% CI, p-value (one-way ANOVA), and Cohen's d. ST, supratuberosity; IT, infratuberosity. Asterisk denotes p < 0.05.

**Supplementary Table S2. Bivariate analysis of factors associated with estimation error for each model**

| **Model / Explanatory variable** | **β** | **R²** | **p (Prob > F)** |
| --- | --- | --- | --- |
| Normalized-angle (ST) — ST distance | 0.0103261 | 0.0221675 | 0.0165* |
| Normalized-angle (ST) — Angle A | −0.0276091 | 0.165495 | <0.0001* |
| Normalized-angle (ST) — Height h | 0.1208291 | 0.1251938 | <0.0001* |
| Normalized-angle (ST) — Tibial slope | 0.0003845 | 9.66E-06 | 0.9603 |
| Normalized-angle (IT) — IT distance | 0.0211124 | 0.0269918 | 0.0081* |
| Normalized-angle (IT) — Angle B | 0.0194110 | 0.0135530 | 0.0614 |
| Normalized-angle (IT) — Height h′ | 0.1933176 | 0.1536709 | <0.0001* |
| Normalized-angle (IT) — Tibial slope | −0.0170459 | 0.0050094 | 0.2564 |
| 1°/1 mm — ST distance | 0.1004592 | 0.4239263 | <0.0001* |
| 1°/1 mm — Angle A | −0.0452240 | 0.0897197 | <0.0001* |
| 1°/1 mm — Height h | 0.5922643 | 0.6077706 | <0.0001* |
| 1°/1 mm — Tibial slope | 0.0136596 | 0.0024624 | 0.4265 |
| 1°/1.4 mm — IT distance | −0.0164045 | 0.0102590 | 0.1039 |
| 1°/1.4 mm — Angle B | 0.0335180 | 0.0254403 | 0.0101* |
| 1°/1.4 mm — Height h′ | 0.0987502 | 0.0252434 | 0.0104* |
| 1°/1.4 mm — Tibial slope | −0.0136491 | 0.0020220 | 0.4712 |
| Osteotomy-depth (ST) — ST distance | 0.0089337 | 0.012842 | 0.0686 |
| Osteotomy-depth (ST) — Angle A | −0.040771 | 0.27933 | <0.0001* |
| Osteotomy-depth (ST) — Height h | 0.177678 | 0.209525 | <0.0001* |
| Osteotomy-depth (ST) — Tibial slope | 0.005586 | 0.001551 | 0.5379 |
| Osteotomy-depth (IT) — IT distance | 0.0038832 | 0.000432 | 0.7391 |
| Osteotomy-depth (IT) — Angle B | 0.103233 | 0.181496 | <0.0001* |
| Osteotomy-depth (IT) — Height h′ | 0.5319052 | 0.550816 | <0.0001* |
| Osteotomy-depth (IT) — Tibial slope | −0.03087 | 0.007691 | 0.1695 |

β, regression coefficient; R², coefficient of determination; p, Prob > F. ST, supratuberosity; IT, infratuberosity. Asterisk denotes p < 0.05.
